# Acute *Plasmodium berghei* Mouse Infection Elicits Perturbed Erythropoiesis With Features That Overlap With Anemia of Chronic Disease

**DOI:** 10.3389/fmicb.2020.00702

**Published:** 2020-04-16

**Authors:** Asha Lakkavaram, Rachel J. Lundie, Hang Do, Alister C. Ward, Tania F. de Koning-Ward

**Affiliations:** ^1^School of Medicine, Deakin University, Waurn Ponds, VIC, Australia; ^2^Infection and Immunity Program, Department of Biochemistry and Molecular Biology, Biomedicine Discovery Institute, Monash University, Clayton, VIC, Australia

**Keywords:** *Plasmodium*, anemia, erythropoiesis, hematopoiesis, STAT5

## Abstract

Severe malaria anemia is one of the most common causes of morbidity and mortality arising from infection with *Plasmodium falciparum*. The pathogenesis of malarial anemia is complex, involving both parasite and host factors. As mouse models of malaria also develop anemia, they can provide a useful resource to study the impact of *Plasmodium* infections and the resulting host innate immune response on erythropoiesis. In this study, we have characterized the bone marrow and splenic responses of the erythroid as well as other hematopoietic lineages after an acute infection of Balb/c mice with *Plasmodium berghei.* Such characterization of the hematopoietic changes is critical to underpin future studies, using knockout mice and transgenic parasites, to tease out the interplay between host genes and parasite modulators implicated in susceptibility to malaria anemia. *P. berghei* infection led to a clear perturbation of steady-state erythropoiesis, with the most profound defects in polychromatic and orthochromatic erythroblasts as well as erythroid colony- and burst-forming units (CFU-E and BFU-E), resulting in an inability to compensate for anemia. The perturbation in erythropoiesis was not attributable to parasites infecting erythroblasts and affecting differentiation, nor to insufficient erythropoietin (EPO) production or impaired activation of the Signal transducer and activator of transcription 5 (STAT5) downstream of the EPO receptor, indicating EPO-signaling remained functional in anemia. Instead, the results point to acute anemia in *P. berghei*-infected mice arising from increased myeloid cell production in order to clear the infection, and the concomitant release of pro-inflammatory cytokines and chemokines from myeloid cells that inhibit erythroid development, in a manner that resembles the pathophysiology of anemia of chronic disease.

## Introduction

Malaria is a global health problem caused by infection with parasites belonging to the genus *Plasmodium*. In 2016, there were ∼216 million episodes of malaria resulting in an estimated 445,000 deaths (WHO, 2017). *Plasmodium falciparum* is responsible for the majority of the disease burden and the most common pathology that arises as a result of infection is moderate to severe malaria anemia. Infection with non-falciparum species can also give rise to anemia. Severe malaria anemia (SMA) is defined as a hemoglobin concentration ≤5 g/dl or hematocrit <15% for children under the age of 12 (or 7 g/dl or hematocrit <20% for adults), in conjunction with a high parasitemia (>10,000 parasites/μl blood) and a normocytic blood film whereas patients with mild anemia have a hemoglobin concentration ≤11 g/dl (WHO Tropical Medicine and International Health, 2014). The anemia of *P. falciparum* malaria is typified by low numbers of red blood cells (RBCs), with a pronounced absence of reticulocytes (Roberts et al., 2005). Thrombocytopenia, splenomegaly, hepatomegaly and jaundice can also be observed in patients with SMA and increased hemolysis and bone marrow suppression is additionally evident (Helleberg et al., 2005).

The pathogenesis of malarial anemia is multi-factorial and both parasite and host-mediated factors play a role [reviewed in Lamikanra et al. (2007)]. Anemia develops rapidly in patients with severe malaria arising from *P. falciparum* infection. Parasitized RBCs (pRBCs) are destroyed as a result of maturation of parasites into schizonts and subsequent rupture from RBCs. The pRBCs are also destroyed by the mononuclear phagocyte system in the spleen, which recognize the presence of foreign parasite antigens on the surface of pRBCs (Patel et al., 2004). Destruction of pRBCs may involve antibody-dependent cell-mediated cytotoxicity (ADCC) (Arora et al., 2016), complement-dependent killing (Boyle et al., 2015) or opsonin-independent killing mechanisms (Su et al., 2002). However, it is the hemolysis of uninfected RBCs that is the more significant contributor to the rapid development of anemia and low hematocrit observed in patients with hyperparasitemia (Looareesuwan et al., 1987; Dondorp et al., 1997) since for every pRBC removed or destroyed by the mononuclear phagocyte system, at least ten uninfected RBCs (uRBCs) are removed from the circulation (Evans et al., 2006).

Alteration to the maturation and/or differentiation of RBCs leading to fewer RBCs leaving the bone marrow is also a contributing factor to anemia (Wickramasinghe and Abdalla, 2000). Moreover, the production of abnormal RBCs (dyserythropoiesis) has also been observed in marrow aspirates taken from patients infected with *P. falciparum* and *Plasmodium vivax* (Abdalla and Wickramasinghe, 1988; Wickramasinghe et al., 1989). However, the mechanisms that give rise to insufficient erythropoiesis and dyserthropoiesis are still poorly defined. In healthy individuals, erythropoiesis is regulated by erythropoietin (EPO) that is secreted by the kidney in response to hypoxia. EPO binds to the EPO-receptor on hematopoietic cells (Youssoufian et al., 1993), leading to the downstream activation of the Janus kinase 2 (JAK2) - Signal transducer and activator of transcription 5 (STAT5) pathway (Witthuhn et al., 1993; Socolovsky et al., 2001) and the differentiation and proliferation of cells within the erythrocytic lineage to generate mature RBCs. Erythropoiesis is also regulated by stem cell factor (SCF) binding to the c-Kit receptor, which in concert with EPO-receptor signaling induces progenitor survival (Dolznig et al., 2001). Clinical evaluation of patients with SMA has revealed this pathology is characterized by significantly lower numbers of erythroid colony-forming cells [specifically the burst forming units (BFU-E)], premature death of erythroblasts and low reticulocytosis (Wickramasinghe et al., 1982; Maggio-Price et al., 1985; Abdalla and Wickramasinghe, 1988; Jootar et al., 1993). It remains unclear whether deficiency of EPO is a major contributor to inadequate erythropoiesis in malaria. In several human studies, EPO production was defective or levels were not as high as would be expected for the degree of anemia observed (Burgmann et al., 1996; El Hassan et al., 1997; Vedovato et al., 1999; Leowattana et al., 2008), however, other studies reported elevated levels of EPO in the serum of African children with severe *P. falciparum*-related anemia (Burchard et al., 1995; Verhoef et al., 2002).

Various parasite mediators such as hemozoin (a by-product of hemoglobin digestion by *Plasmodium*) (Pagola et al., 2000) and glycosylphosphatidylinositols (GPIs) attached to parasite antigens have been shown to affect erythropoiesis during a *Plasmodium* infection. Hemozoin promotes premature apoptosis of erythroid precursors (Lamikanra et al., 2009) and causes dysregulation in the innate immune response (Perkins et al., 2011), influencing the production of pro-inflammatory cytokines and chemokines. Indeed, the balance between pro- and anti-inflammatory cytokines is likely to be a critical determinant of anemia. Several of these cytokines, such as TNF-α and IFN-γ play a protective role in malaria by arresting parasite replication and enhancing parasite killing (Clark et al., 1990), but also contribute to anemia by inhibiting erythropoiesis (Miller et al., 1989; Dufour et al., 2003).

To date the effect of *Plasmodium* infection on erythropoiesis has been studied in a variety of different contexts, including in patients, using *in vitro* erythroblast infections, and in various mouse models of *Plasmodium* infection and anemia [for a review, see Lamikanra et al. (2009)]. However, there are limited studies examining the interplay between the erythrocytic and the hematopoietic compartments more broadly in response to a *Plasmodium* infection, which together influence whether optimal pro-inflammatory and erythropoietic responses can be mounted to fight infection and prevent anemia. In this study, we examined the erythropoietic and wider hematopoietic responses that occur in the bone marrow and spleen of Balb/c mice after an acute infection with *Plasmodium berghei*, a rodent malaria strain which is most widely used for gene knockout studies. Such characterization is critical to underpin future studies to tease out and validate the interplay between parasite modulators and host genes that determine susceptibility to malaria anemia that have, for example, been identified through human genome wide association studies.

## Materials and Methods

### Mice and Ethics Approval

In this study, female Balb/c mice of 6–8 weeks of age were used as donor and recipient mice for *P. berghei* infection. Mice were housed under controlled conditions at 21°C temperature with a 12:12 h light:dark cycle. All experiments involving the use of animals were performed in strict accordance with the recommendations of the National Health and Medical Research Council Australian ‘Code of practice for the care and use of animals for scientific purposes.’ The protocols were approved by the Deakin University Animal Welfare Committee (approval numbers G37/2013 and G08/2017).

### Infection of Mice With *P. berghei*

Mice were infected with wild-type *P. berghei* ANKA and *P. berghei* EXP2-2A-FRT, the latter expressing an exported GFP reporter (KAHRP_L_-GFP), in which the sequence of KAHRP (*P. falciparum* knob-associated histidine-rich protein) that directs export into the host RBC is fused in frame to GFP, under the transcriptional control of the HSP70 promoter (Kalanon et al., 2016). For infection studies, 10^6^ parasitized RBCs (pRBCs) were injected into the intraperitoneal cavity (*n* = 6 mice/group). Infections were initiated on either day 0 (d0) or 3 days later (d3) and parasitemia was determined daily by counting Giemsa-stained blood smears, with a minimum of 1,000 RBC counted. On the 8th day, all mice (thus infected for either 8 days or 5 days, respectively) as well as control uninfected mice were humanely killed.

### Mouse Blood Analysis

For measuring hematological parameters such as hemoglobin and hematocrit, 20 μl mouse blood was collected using minivettes (Sarstedt^TM^) and analyzed using a hematology analyzer according to the manufacturer’s instructions (SCIL).

### Harvesting of Mouse Bone Marrow and Spleen Cells

Bone marrow cells were extracted from both the femurs and tibias of uninfected and *P. berghei*-infected mice by flushing out the marrow with 1 ml RPMI 1640 media (Life Technologies) using a 26-gauge needle. Spleen cells were isolated by mashing the entire spleen in 5 ml RPMI 1,640 media and passing the cells through a 40 μm nylon mesh cell strainer (Interpath). The cells were centrifuged at 1,000 × *g* and the pellet was resuspended in PBS buffer containing 1% (w/v) bovine serum albumin (BSA).

### Antibody Staining for Flow Cytometric Analysis

Bone marrow or spleen cells at 10^6^ cells/50 μl were incubated with anti-mouse CD16/CD32 (Fcγ III/II Receptor; 2.5 μg/10^6^ cells) for 15 min on ice. Samples were subsequently stained with PE-conjugated anti-mouse Ter-119 (0.5 μg/10^6^ cells), APC-conjugated anti-mouse CD44 (IM7; 0.2 μg/10^6^ cells), APC-Cy7-conjugated anti-CD11b (M1/70; 0.1 μg/10^6^ cells), FITC-conjugated anti-GR-1 (RB6-8C5; 0.1 μg/10^6^ cells), PE-conjugated anti-Ly6G (1A8; 0.1 μg/10^6^ cells), BV421-conjugated anti-CD11c (N418; 0.2 μg/10^6^ cells) on ice for 20–30 min in the dark. Cells were pelleted at 1,000 × *g*, washed in PBS containing 1% (w/v) BSA and resuspended in 0.2 ml of PBS containing 1% (w/v) BSA. The cells were then incubated with 7-AAD (2 μl/1 × 10^6^) cells for 10 min to allow live/dead discrimination prior to FACS analysis.

For analysis of STAT5 activation, bone marrow or spleen cells were first pelleted at 1,000 × *g* at 4°C and resuspended in 1 ml of phosphowash buffer [1 × PBS, 1 mM sodium orthovanadate, 1 mM β-glycerol phosphate and 100 mM sodium fluoride containing 1 × PhosSTOP inhibitors (Sigma)]. To this buffer, PE-conjugated anti-Ter119 and APC-conjugated anti-CD44 in conjunction with VS-450 were added as above and samples incubated for 30 min on ice. The cells were then treated with 30 international units (I.U) of erythropoietin (EPO) (Janssen, United Kingdom) at 37°C for 30 min. Following this, the cells were pelleted and resuspended in fixing buffer (2% (v/v) paraformaldehyde and 0.0075% (v/v) glutaraldehyde in PBS containing 3% (w/v) BSA) for 10 min at room temperature (RT), before quenching with phosphowash buffer containing 500 mM Tris for 10 min at RT, washing with phosphowash buffer, and then permeabilization with phosphowash buffer containing 0.2% (v/v) Triton-X for 10 min at RT. The cells were washed with phosphowash buffer and resuspended in 100 μl phosphowash buffer containing either AF488-conjugated anti-phospho-STAT5 antibody (pY694) or the isotype control AF488-conjugated anti-mouse IgG_1_, κ (20 μl/10^6^ cells). After incubation for 30 min on ice, cells were washed twice and resuspended in 200 μl phosphowash buffer for FACS analysis.

### Flow Cytometry and Cell Sorting

A minimum of 10,000 and up to 10,000,000 stained cells were analyzed by flow cytometry with a BD FACS CANTO II flow cytometer (BD Biosciences) equipped with blue (488 nm, 20 mW), red (633 nm, 17 mW), and violet (405 nm, 30 mW) lasers. In circumstances where cells were sorted, a BD FACS Aria II cell sorter, equipped with violet (405 nm, 50 mW), blue (488 nm, 20 mW), and red (633 nm, 18 mW) lasers, was used. Events were collected using FACS Diva software or BD FACS software. Cell debris and noise were removed from gating analysis based on FSC/SSC properties. Single cells were gated based on the FSC area to height ratio. Live/dead cell discrimination was achieved using 7-AAD or VS-450 and only live cells were analyzed further. Compensation and further analysis was performed using FlowJo v.10.0.6 (Tree Star).

### Methyl Cellulose Colony Assay for Mouse Hematopoietic Progenitor Cells

Bone marrow cells were harvested aseptically from femurs and tibias of mice using Iscove’s modified Dulbecco’s medium (IMDM) (Sigma) containing 2% (v/v) FBS and resuspended to a final concentration of 5 × 10^5^ cells/100 μl IMDM. To this cell suspension, 1 ml of methyl cellulose (R&D systems) was added and mixed by vortexing before being spread onto a 5 ml cell culture dish and incubated at 37°C with 5% CO_2_. On days 4 and 8 after plating, colonies were enumerated using an inverted microscope (40× magnification) and identified as either CFU-GEMM (colony forming unit-granulocyte, erythrocyte, monocyte, and megakaryocyte), CFU-GM (colony forming unit-granulocyte and macrophage), CFU-G (colony forming unit-granulocyte) CFU-M (colony forming unit-macrophage), CFU-E (colony forming unit, erythroid), or BFU-E (burst forming unit, erythroid) based on their morphology. The numbers of CFU-Meg (CFU-megakaryocytes) could not be determined as their growth is not supported by the methyl cellulose used in the assay.

### Microscopy

Peripheral blood smears stained with Giemsa were visualized by light microscopy at 100× (NA 1.25) magnification under oil. Cells from the methyl cellulose colony assay were visualized by light microscopy at 40× (NA 0.95) magnification. Erythroblasts parasitized with GFP-expressing parasites were labeled with PE-conjugated anti-mouse Ter119 and the nuclear stain 4′,6-diamidino-2-phenylindole (DAPI, 500 ng/nl) (Vector Labs) and visualzed on an Olympus IX71 inverted wide field microscope equipped with DAPI (Excitation 330–385/emission 420), GFP (470–595/510–550) and DsRed (545–580/610IF) filter cubes at 100× (NA 1.37) magnification under oil. Images were processed using ImageJ version 1.47d^1^.

### Serum Cytokine Analysis

Mouse serum was obtained by centrifuging mouse blood that had been allowed to clot at RT for 30 min at 5,000 × *g* for 10 min at 4°C and harvesting the supernatant. Cytokine levels in the serum were measured using the ProcartaPlex Mouse Cytokine and Chemokine Panel 1 kit (26 plex, eBioscience) according to manufacturer’s instructions. The cytokines and chemokines analyzed were IL-12, IL-23, IL-27, CCL2 (MCP-1), CCL5 (RANTES), CCL7 (MCP-3), CXCL1 (GRO-α), CXCL10 (IP-10), Eotaxin, GM-CSF, IFN-γ, IL-1β, IL-10, IL-13, IL-17A, IL-18, IL-2, IL-22, IL-4, IL-5, IL-6, IL-9, MIP-1α, MIP-1β, TNF-α, and MIP-2. Serum EPO levels were separately quantified using a mouse EPO ELISA kit (Sigma-Aldrich) according to manufacturer’s instructions.

### Statistical Analysis

Statistical analysis was performed using Graph Pad prism v6 (GraphPad Software, La Jolla, CA, United States). For all experiments, an unpaired Student’s *t*-test was used to compare two groups. A *p*-value < 0.05 was considered significant.

## Results

### Changes to the Cellularity of the Bone Marrow and Spleen During a *P. berghei* Infection

**FIGURE 1:**
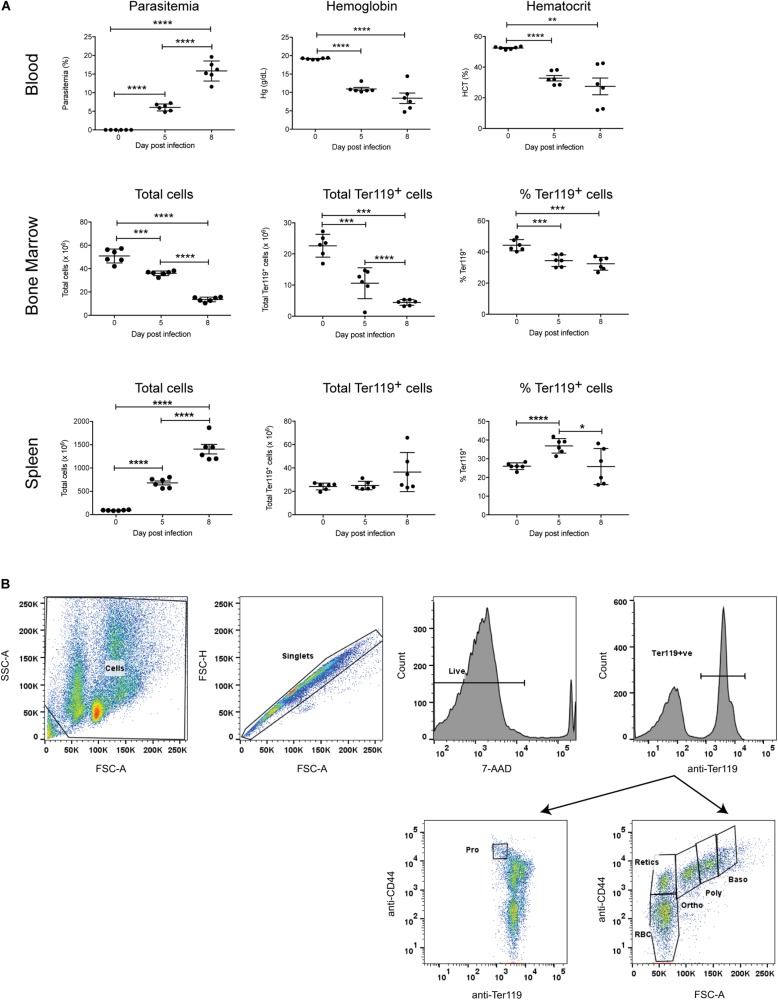
Global impact of *Plasmodium berghei* infection. The global impact of malaria infection on blood parameters was measured **(A)**, including blood parasitemia as measured by examination of blood smears, hemoglobin levels and hematocrit using a hematology analyzer (top panel), as well as total cells, total Ter119^+^ cells and percentage Ter119^+^ cells in the bone marrow and spleen (middle and bottom panels, respectively), by FACS analysis. Plotted are results from individual mice as well as mean ± SD. An unpaired *t*-test was used to calculate statistical significance between two groups: *p < 0.05, **p < 0.01, ***p < 0.001, ****p < 0.0001. The FACS gating strategy for identifying Ter119^+^ cells and distinct stages of erythroid differentiation in the bone marrow and spleen described is outlined **(B)**. Cells were gated on size and granularity based on forward and side scatter to avoid debris. After selection of singlets, 7-AAD-negative live cells and Ter119 (PE-positive) erythroid cells, distinct stages of erythroid differentiation were identified and quantified, using CD44 expression and cell size. Shown is example of cells isolated from the bone marrow of uninfected Balb/c mice. Pro, proerythroblasts; Baso, basophilic erythroblasts; Poly, polychromatic erythroblasts; Ortho, orthochromatic erythroblasts; Retics, reticulocytes; RBC, red blood cells. Other populations outside the areas gated remain undefined (Chen et al., 2009).

To investigate the erythropoietic response to acute *P. berghei* infection, groups of female Balb/c mice (*n* = 6) were infected with 10^6^ *P. berghei*-parasitized RBCs (pRBCs). As expected, significant parasitemia was observed, with a mean parasitemia of 6.1% at 5 dpi, increasing to 15.8% at 8 dpi (Figure 1A). Mice were anemic by 5 dpi, based on significant decreases in their hemoglobin levels and hematocrit (Figure 1A). Nevertheless, the RBCs remained normocytic as determined by measuring the mean corpuscular volume (0 dpi, 17.40 ± 0.15; 7 dpi, 17.43 ± 1.09) and mean corpuscular hemoglobin (0 dpi, 46.33 ± 0.53; 7 dpi, 49.05 ± 0.97). *P. berghei*-infected mice showed a dramatic decrease in bone marrow cellularity, whilst the total number of cells in the spleen markedly increased over the course of infection (Figure 1A), consistent with the splenomegaly observed in these mice (data not shown).

### Changes to the Erythroid Populations in the Bone Marrow and Spleen

To specifically examine the erythroid lineage in the bone marrow and spleen, Ter119 was used as a marker for erythroid cells at all stages of differentiation, from early proerythroblasts to the mature erythrocyte stage (Kina et al., 2000). The FACS gating strategy used for the analysis is outlined in Figure 1B. Both the total number and relative proportion of Ter119^+^ cells in the bone marrow significantly decreased during infection (Figure 1A). Conversely, there were no significant differences in the total number of Ter119^+^ cells in the spleen at either 5 dpi or 8 dpi, however, the proportion of Ter119^+^ cells relative to other cells in the spleen did significantly increase at 5 dpi (Figure 1A).

**FIGURE 2:**
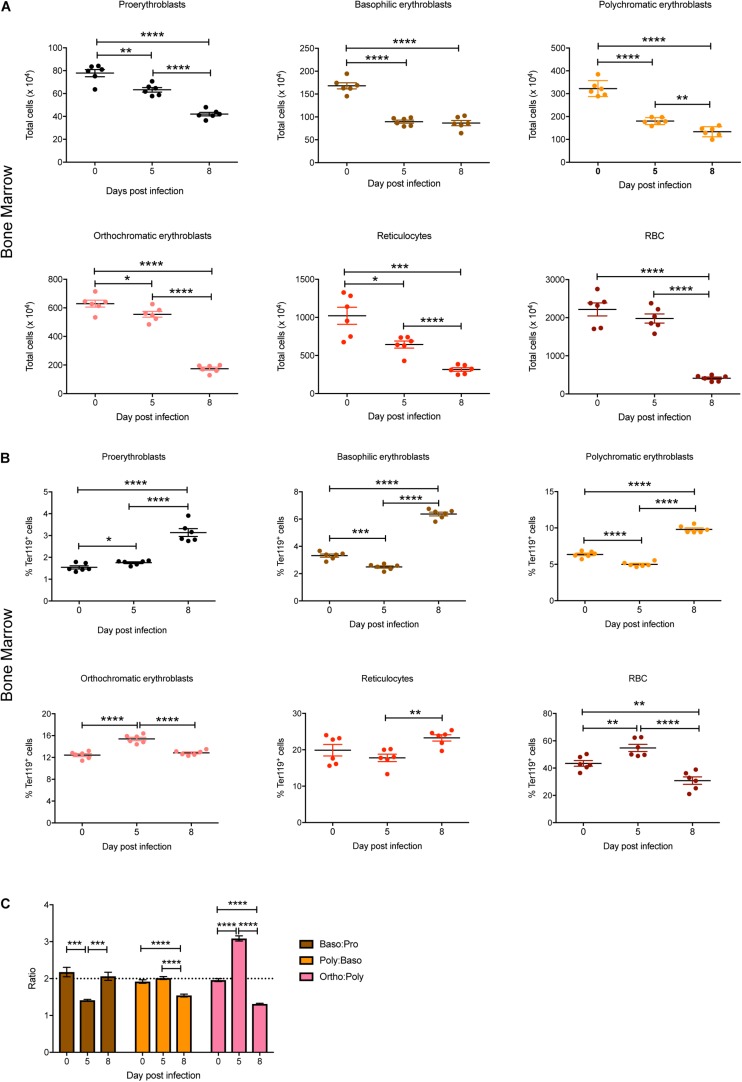
Impact of *P. berghei* infection on erythroblast maturation dynamics in the bone marrow. Analysis of the total number **(A)** and proportion of Ter119^+^ cells **(B)** of the indicated stages of erythrocyte maturation in the bone marrow during a *P. berghei* infection using the gating strategy outlined in Figure 1B. Plotted are the results from individual mice as well as mean ± SD (*n* = 6 mice). From the data in **(B)**, the ratio of the indicated erythrocyte populations was determined, with mean ± SD shown **(C)**. An unpaired *t*-test was used to calculate statistical significance between two groups: *p < 0.05, **p < 0.01, ***p < 0.001, ****p < 0.0001.

Individual stages of erythroid maturation (proerythroblasts, basophilic erythroblasts, polychromatic erythroblasts, orthochromatic erythroblasts, reticulocytes, and RBCs) were next analyzed based on size and expression of CD44 and Ter119 as described (Chen et al., 2009) (see Figure 1B for gating strategy and Supplementary Figure S1 for representative FACS plots at 0, 5, and 8 dpi). All erythroid stages except RBCs were significantly decreased in the bone marrow by 5 dpi, with all of these except basophilic erythroblasts decreasing further by 8 dpi, by which time the mature RBCs were also significantly reduced (Figure 2A). Analysis of the relative proportions of the various erythroid stages in the bone marrow revealed that by 5 dpi, basophilic and polychromatic erythroblast populations were significantly reduced, while the proportions of proerythroblasts, orthochromatic, and mature RBCs were significantly increased (Figure 2B). At 8 dpi, the proportion of mature RBCs was significantly decreased relative to proerythroblasts, basophilic and polychromatic erythroblasts and reticulocytes (Figure 2B).

During normal steady-state erythropoiesis, a ratio of 1 proerythroblast (pro): 2 basophilic erythroblasts (baso): 4 polychromatic erythroblasts (poly): 8 orthochromatic erythroblasts (ortho) is typically observed (Chen et al., 2009), as seen in uninfected mice (Figure 2C). At 5 dpi, the baso: pro ratio was significantly decreased in the bone marrow, while the ortho:poly ratio was significantly increased (Figure 2C). By 8 dpi the low baso:pro ratio had returned to normal levels, however, the poly:baso and ortho:poly ratios were significantly decreased (Figure 2C).

**FIGURE 3:**
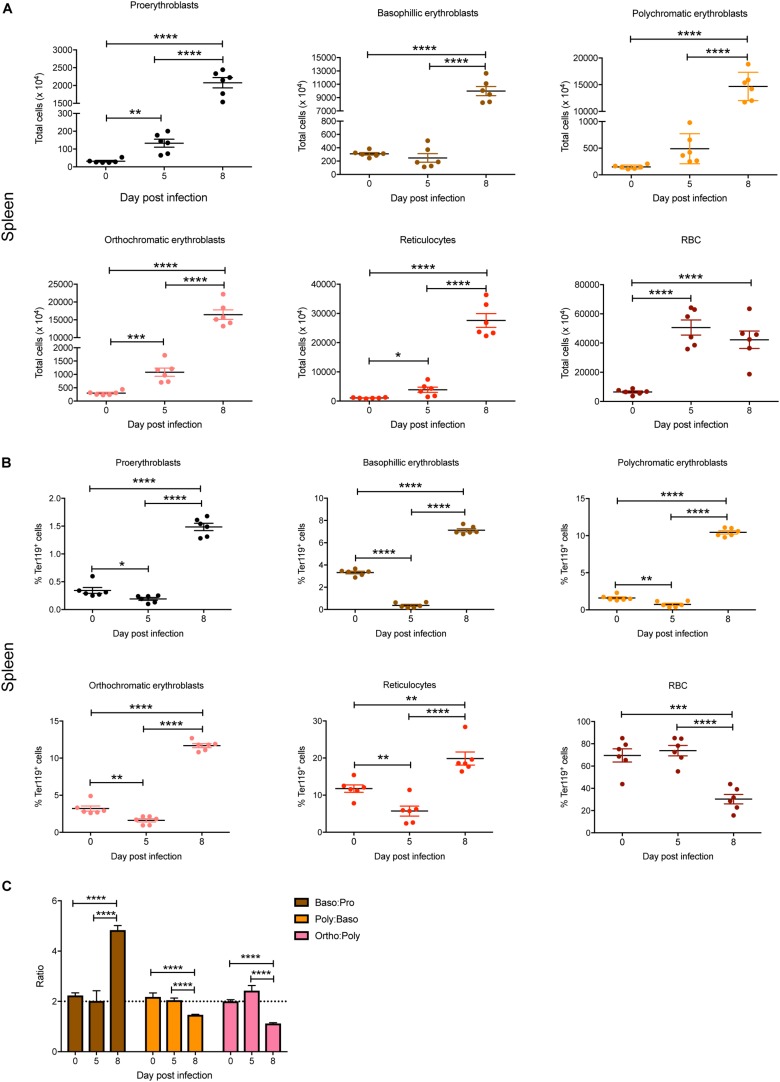
Impact of *P. berghei* infection on erythroblast maturation dynamics in the spleen. Analysis of the total number **(A)** and proportion of Ter119^+^ cells **(B)** of the indicated stages of erythrocyte maturation in the spleen during a *P. berghei* infection using the gating strategy outlined in Figure 1B. Plotted are the results from individual mice as well as mean ± SD (*n* = 6 mice). From the data in **(B)**, the ratio of erythroblast populations was determined with mean ± SD shown **(C)**. An unpaired *t*-test was used to calculate statistical significance between two groups: *p < 0.05, **p < 0.01, ***p < 0.001, ****p < 0.0001.

In the spleen, total numbers of all erythroid stages were significantly increased by 5 dpi except for basophilic and polychromatic erythroblasts (Figure 3A). By 8 dpi, significant increases in total cell numbers were observed for all erythroid stages, with the exception of mature RBC, which showed no significant changes between 5 and 8 dpi (Figure 3A). Interestingly, although the proportions of all blast populations and reticulocytes in the spleen were significantly reduced on 5 dpi, by 8 dpi significant increases were observed for all populations except for mature RBCs, which were significantly decreased, even below naïve levels (Figure 3B). Erythroblast ratios were not significantly altered until 8 dpi, at which time the baso:pro ratio was significantly increased whereas the poly:baso and ortho:poly ratios were significantly decreased (Figure 3C).

### *Plasmodium berghei* Does Not Infect Erythroblasts

*Plasmodium berghei* parasites are known to preferentially invade reticulocytes in the blood (Cromer et al., 2006), thereby directly interfering with their differentiation. To investigate whether *P. berghei* can also infect erythroblasts and modulate their maturation, Balb/c mice were infected with *P. berghei* EXP2-2A-FRT parasites expressing a GFP reporter (Pb-GFP) that is exported into the host cell (Kalanon et al., 2016), thereby enabling identification of parasite-infected cells. Spleens were harvested from mice infected with Pb-GFP parasites at 8 dpi and cells were analyzed for Ter119 and GFP co-expression by flow cytometry. Erythroblasts were also sorted based on CD44 and Ter119 expression and then stained with DAPI prior to microscopy. Whilst GFP expression could be readily detected in reticulocytes, this was not the case with erythroblasts, with very few erythroblasts expressing even a weak GFP signal (Supplementary Figures S2A,B). Thus, the ability of *P. berghei* to infect erythroblasts is unlikely to be a significant factor in the perturbation of erythroblast maturation.

### Infected Mice Show Increased Serum EPO Levels

**FIGURE 4:**
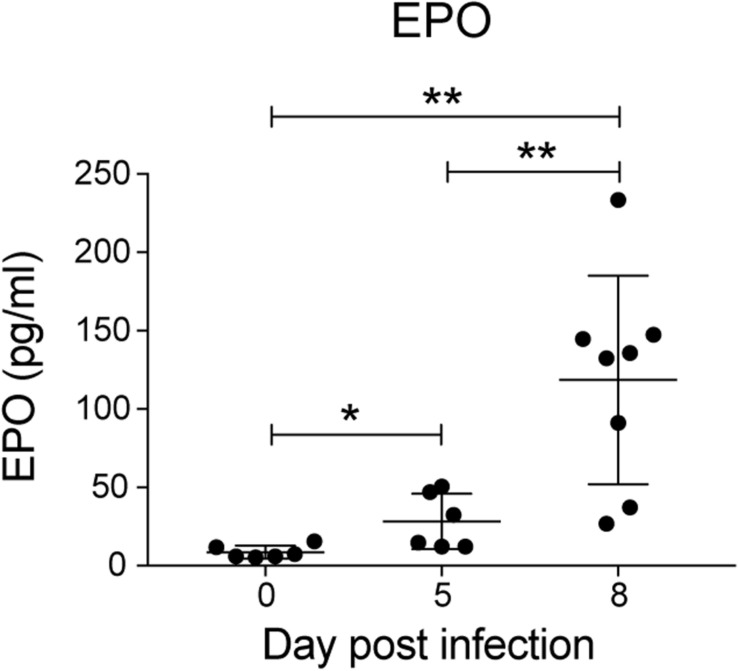
Analysis of erythropoietin levels during *P. berghei* infection. Levels of EPO (pg/ml) in the serum of mice during a *P. berghei* infection was quantified using an EPO ELISA kit. Plotted are the results from individual mice as well as mean ± SD (*n* = 6–8 mice). An unpaired *t*-test was used to calculate statistical significance between two groups: *p < 0.05, **p < 0.01.

Erythropoietin is a key mediator of emergency erythropoiesis in response to low oxygen tension (Koury et al., 1989), therefore another mechanism by which the perturbation in erythropoiesis could be mediated is through defective EPO production. Serum EPO levels significantly increased during *P. berghei* infection (Figure 4), which correlated with the reduced hematocrit to an extent. This indicates that the inefficient erythropoiesis in infected mice was not due to an overt deficiency in EPO production.

### EPO-Mediated STAT5 Activation in Erythroblasts Is Not Altered During Infection

**FIGURE 5:**
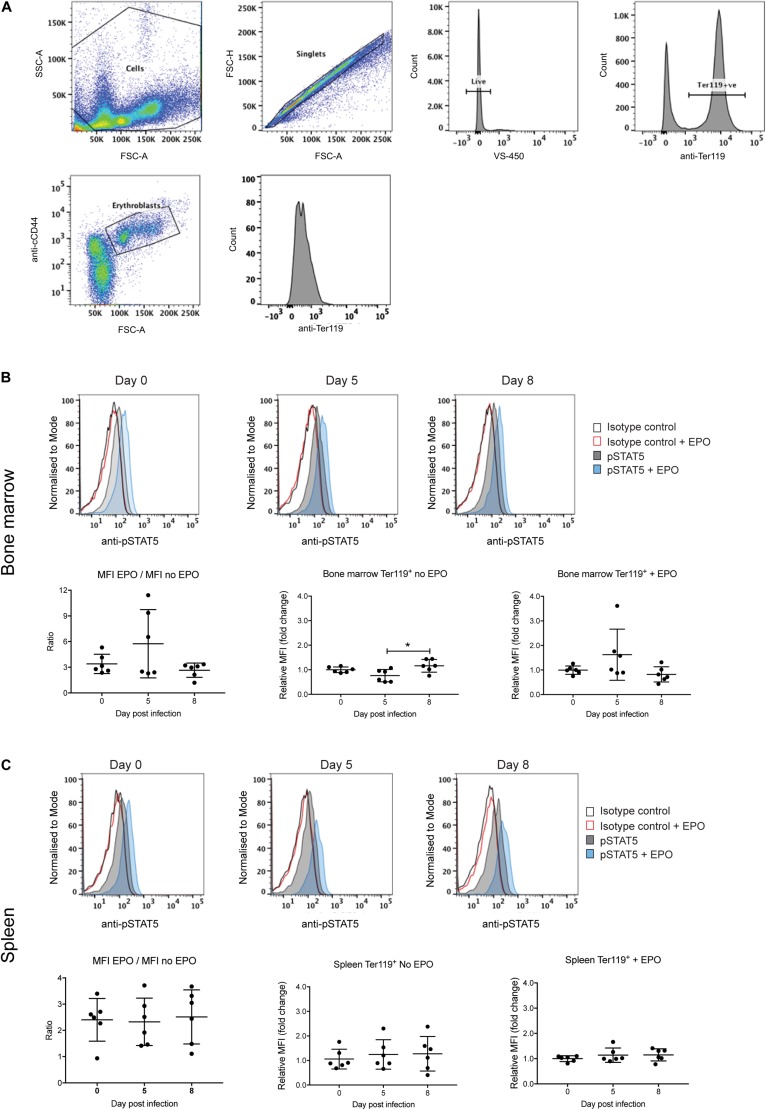
Analysis of STAT5 activation during *P. berghei* infection. Gating strategy to detect pSTAT5 responses in erythroblasts **(A)**. Fixed and permeabilized spleen cells following removal of debris (i) were stained with APC-conjugated CD44, PE-conjugated Ter119 and AF488-conjugated pSTAT5 antibodies and 7-AAD. After selection of singlets, live cells and Ter119^+^ cells, erythroblasts were gated based on CD44 expression and cell size and pSTAT5 staining quantified. STAT5 activation in Ter119^+^ erythroblasts from the bone marrow **(B)** and spleen **(C)** before and after *ex vivo* EPO stimulation as measured by FACS with anti-pSTAT5. Shown in the upper panels are representative medium intensity histograms of samples analyzed with anti-pSTAT5 compared to an isotype control in the presence or absence of EPO stimulation, as indicated. Data in the lower panels represents the ratio of MFI from cells stimulated with EPO to unstimulated cells as well as the fold-change ± EPO from individual mice as well as the mean ± SD (*n* = 6 mice). An unpaired *t*-test was used to calculate statistical significance between two groups. *p < 0.05.

Erythropoiesis could also be impacted by altered signaling by the EPO receptor. Expression of the EPO receptor peaks at the CFU-E and proerythroblast stage and decreases with further blast maturation such that by the reticulocyte stage expression is undetectable (Broudy et al., 1991). Signal transducer and activator of transcription 5 (STAT5) is a key downstream target of EPO receptor signaling, being responsible for the transcriptional activation of many key genes involved in erythropoiesis (Gillinder et al., 2017). Therefore, levels of phosphorylated STAT5 (pSTAT5) were used as a measure of receptor activation in the Ter119^+^ erythroblast population from the bone marrow and spleen, either directly or after EPO stimulation *ex vivo*. The gating strategy to obtain erythroblasts is outlined in Figure 5A. An isotype control antibody was used in place of the anti-phospho STAT5 antibody to control for non-specific FITC labeling of erythroblasts.

Levels of pSTAT5 in unstimulated bone marrow and splenic erythroblasts harvested from uninfected and *P. berghei*-infected mice were low, as indicated by only subtle increases in anti-pSTAT5 labeling compared to the isotype control antibody (Figures 5B,C, upper panels). After stimulation with EPO, pSTAT5 activity increased in all cell populations relative to cells that had not been stimulated with EPO (Figures 5B,C, upper panels). However, no significant differences were observed in the fold-change of pSTAT5 levels in bone marrow or splenic erythroblasts from infected mice relative to uninfected mice, either before or after EPO stimulation (Figures 5B,C, lower panels). This indicates that perturbed EPO receptor signaling is not responsible for the lack of erythroblast differentiation observed in *P. berghei*-infected mice.

### Effect of *P. berghei* Infection on Other Hematopoietic Populations in the Bone Marrow and Spleen

**FIGURE 6:**
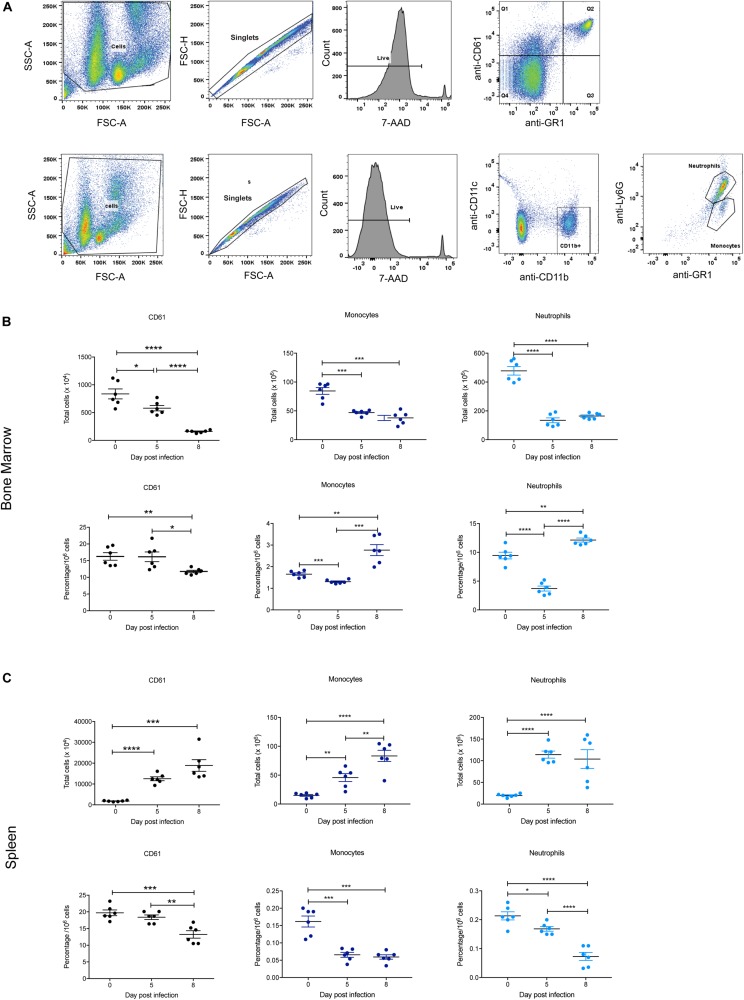
Impact of *P. berghei* infection on megakaryocyte, neutrophil, and monocyte populations in the bone marrow and spleen. Gating strategy for identifying megakaryocytic, monocyte, and neutrophil cells **(A)**. For megakaryocytic cells (top panel), cells were firstly gated on size and granularity based on forward and side scatter to remove debris. FSC-H and FSC-A plots were then used to identify singlets. Only live cells, which are negative for 7-AAD staining were analyzed for CD61 and GR1 staining. Cells positive for CD61 but negative for Gr1 (i.e., those in Q1) were gated and analyzed. Shown is example of cells isolated from the bone marrow of uninfected Balb/c mice. Gating strategy for monocytes and neutrophils (bottom panel). Cells were gated on size and granularity based on forward and side scatter to remove debris. After selection of singlets and live cells, cells were analyzed for CD11c and CDl1b staining. Cells positive for CD11b but negative for CD11c were gated on to remove dendritic cells and the resulting cell population was analyzed for Gr1 and Ly6G expression to distinguish between neutrophils (Gr-1^+^, Ly6G^+^) and monocytes (Gr-1^+^, Ly6G^–^). Shown is example of cells isolated from the bone marrow of uninfected Balb/c mice. CD61^+^ megakaryocytes, CD11b^+^Gr1^+^Ly6G^–^ monocytes and CD11b^+^Gr1^+^Ly6G^+^ neutrophils in the bone marrow **(B)** and spleen **(C)** during the course of a *P. berghei* infection were then examined, from which the total number (top panel) and proportion of cells (lower panel) were calculated. Plotted are the results from individual mice as well as mean ± SD (*n* = 6 mice). An unpaired *t*-test was used to calculate statistical significance between two groups: *p < 0.05, **p < 0.01, ***p < 0.001, ****p < 0.0001.

The BFU-E erythroid lineage is derived from the same common hematopoietic progenitor cell (CFU-GEMM) that yields CFU-Meg (megakaryocyte precursors) and CFU-GM (precursors to CFU-G and CFU-M from which neutrophils and monocytes, respectively, are derived). Therefore, these populations in the bone marrow and spleen were also examined during *P. berghei* infection to provide a global view of hematopoiesis, with the gating strategy outlined in Figure 6A. Representative FACS plots at 0, 5, and 8 dpi are shown in Supplementary Figure S3.

Similar to the Ter119^+^ population, the total number of CD61^+^ megakaryocytic cells significantly decreased in the bone marrow at 5 dpi, with even fewer cells present at 8 dpi (Figure 6B). In contrast, megakaryocytic cell numbers significantly increased in the spleen over the timecourse (Figure 6C). The proportion of CD61^+^ cells in both the bone marrow and spleen were significantly decreased at 8 dpi (Figures 6B,C), indicating thrombocytopenia with late infection.

In keeping with the changes to overall cellularity of the bone marrow in response to infection, total numbers of monocytes and neutrophils in the bone marrow significantly decreased by 5 dpi (Figure 6B). The proportions of monocytes and neutrophils in the bone marrow were also significantly lower at 5 dpi compared to uninfected mice. However, at 8 dpi the proportions of these cell types had rebounded to levels significantly higher than at 0 dpi (Figure 6B). In contrast, the total numbers of monocytes and neutrophils increased significantly in the spleen during infection (Figure 6C). However, the proportion of monocytes in the spleen was significantly reduced at 5 dpi to a level that was maintained at 8 dpi, whilst the proportion of neutrophils was significantly reduced at 5 dpi, and notably more so at 8 dpi (Figure 6C).

### Differentiation of Hematopoietic Cells in the Bone Marrow During Infection

**FIGURE 7:**
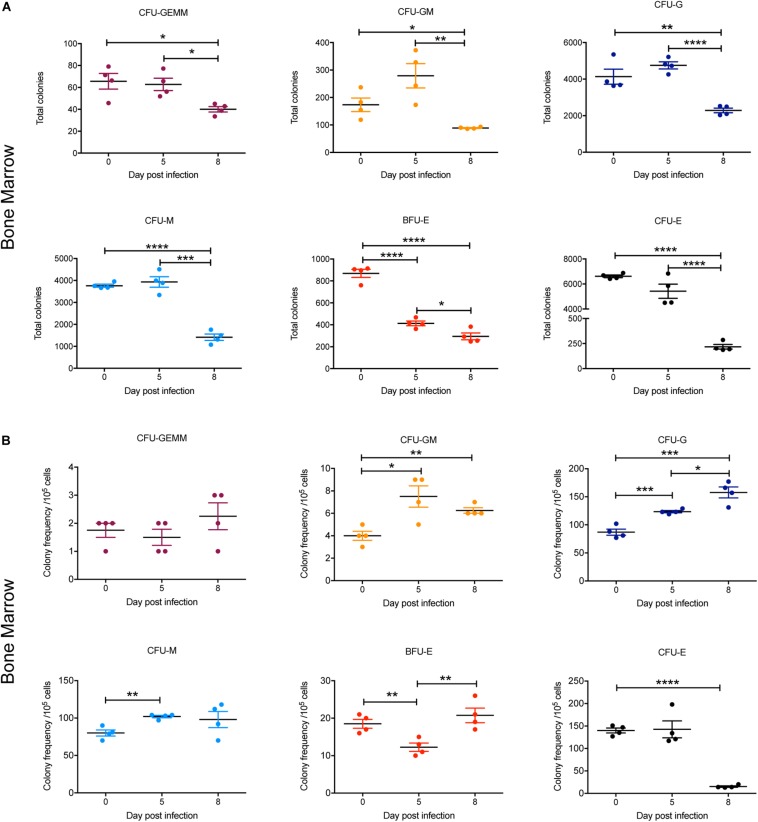
Impact of *P. berghei* infection on colony forming units in the bone marrow. Analysis of the total number **(A)** and the proportion **(B)** of CFUs present in the bone marrow, which were determined by assessing the morphology of colonies of bone marrow cells by microscopy after incubation in medium containing methyl cellulose and relevant cytokines. Data represents the mean ± SD (*n* = 4 independent experiments). An unpaired *t*-test was used to calculate statistical significance between two groups: *p < 0.05, **p < 0.01, ***p < 0.001, ****p < 0.0001.

To gain further insight into observed alterations in the erythroid, megakaryocyte and myeloid lineages during the course of infection, early progenitors in bone marrow and spleen were analyzed using a colony forming unit (CFU) assay. At 5 dpi, the total numbers of all CFUs in the bone marrow were not significantly different to 0 dpi, with the exception of BFU-E, which was significantly decreased twofold (Figure 7A). By 8 dpi, the total number of CFUs in the bone marrow was significantly decreased for all colony types, with CFU-E showing the greatest decline (∼30-fold) (Figure 7A). However, when the relative frequencies of the different CFUs per 10^5^ cells was determined, the proportion of CFU-GM, CFU-G, and CFU-M was significantly increased at 5 dpi and remained significantly higher for CFU-GM and CFU-G at 8 dpi (Figure 7B). In contrast, the proportion of BFU-E was significantly decreased at 5 dpi but had returned to pre-infection levels by 8 dpi, whilst CFU-E was significantly decreased by this stage. CFU-GEMM did not change during infection (Figure 7B).

**FIGURE 8:**
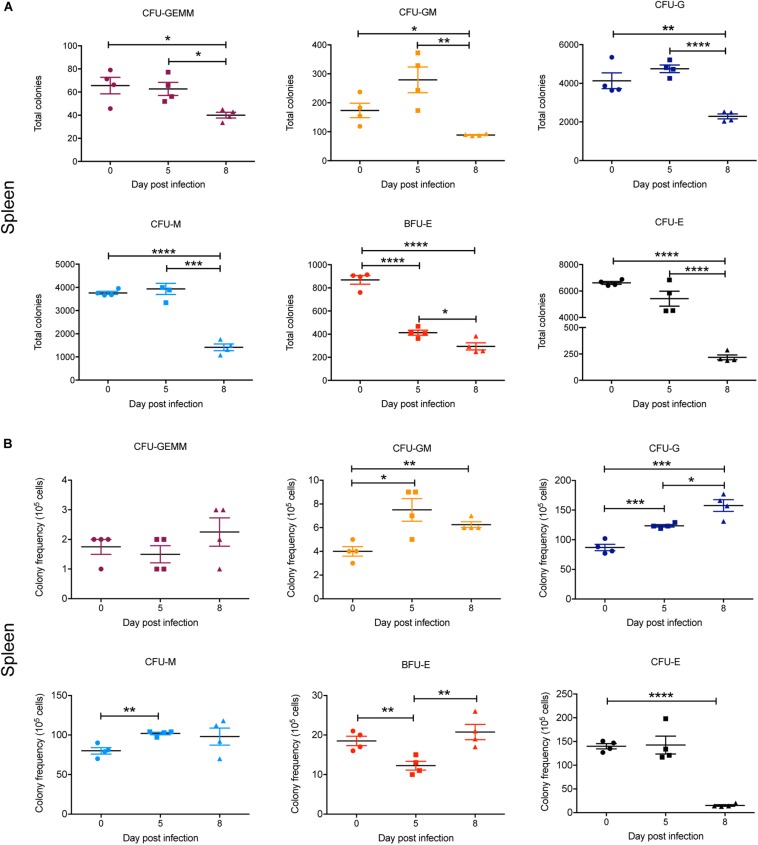
Impact of *P. berghei* infection on colony forming units in the spleen. Analysis of the total number **(A)** and the proportion **(B)** of CFUs present in the spleen, which were determined by assessing the morphology of colonies of spleen cells by microscopy after incubation in medium containing methyl cellulose and relevant cytokines. Data represents the mean ± SD (*n* = 4 independent experiments). An unpaired *t*-test was used to calculate statistical significance between two groups: *p < 0.05, **p < 0.01, ***p < 0.001, ****p < 0.0001.

In the spleen, there was a marked and statistically significant increase in the total numbers of all CFUs by 5 dpi and, with the exception of CFU-GEMM, all populations further increased by 8 dpi (Figure 8A). Similarly, the frequency of all CFUs in the spleen was increased at 5 dpi, except for CFU-GEMM and CFU-GM, with the latter significantly decreasing. However, only CFU-G had increased further by 8 dpi, with CFU-GM decreasing further and both BFU-E and CFU-E frequency decreasing although remaining higher than at 0 dpi (Figure 8B).

### Infected Mice Show Increased Serum Levels of Proinflammatory Cytokines and Chemokines

**FIGURE 9:**
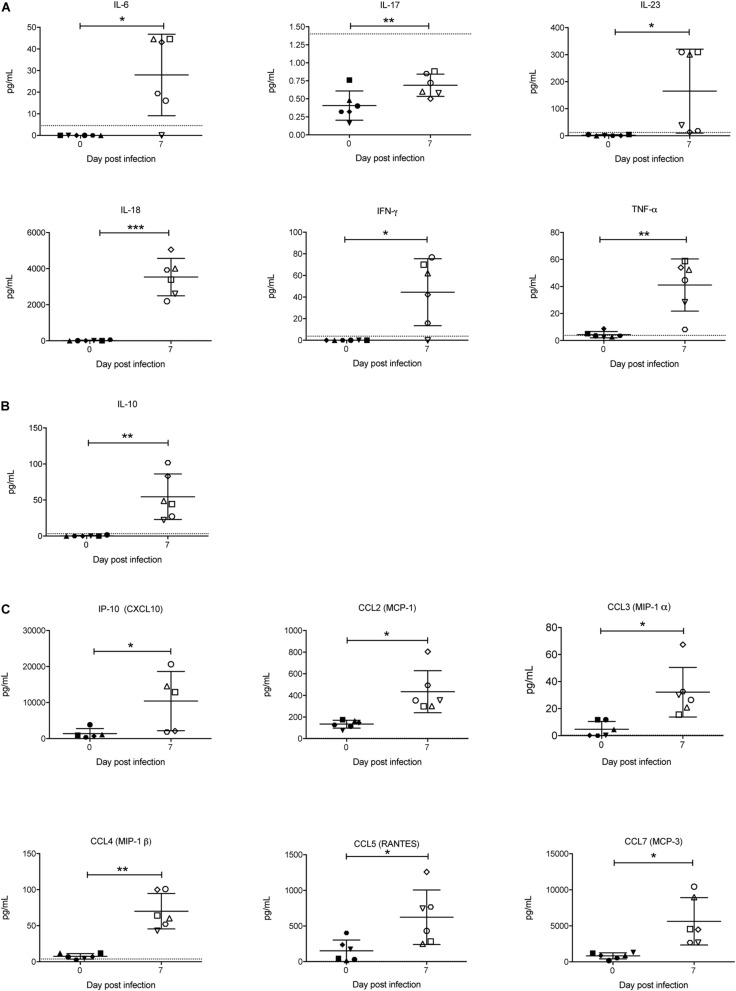
Analysis of pro-inflammatory and anti-inflammatory cytokines and chemokines during *P. berghei* infection. Levels of proinflammatory cytokines **(A)**, anti-inflammatory cytokines **(B)**, and chemokines **(C)** were measured with a ProcartaPlex Mouse Cytokine and Chemokine Panel 1 kit and those that were significantly upregulated in the serum of infected mice at 7 dpi are shown. The lower limits of detection are indicated by a dotted line. Data represents results from individual mice as well as the mean ± SD (*n* = 6 mice). An unpaired *t*-test was used to calculate statistical significance: *p < 0.05, **p < 0.01, ***p < 0.001.

Cytokines are known to play a key role in determining the outcome of a *Plasmodium* infection (i.e., pathogenesis or protection) (Angulo and Fresno, 2002). Therefore, the concentration of a range of cytokines in the sera was also examined. Of the pro-inflammatory cytokines, IFN-γ, TNF-α, IL-6, IL-18, and IL-23 were significantly increased in response to *P. berghei* infection (Figure 9A). Although IL-17 levels were below the lower limit of quantification, they also increased upon infection (Figure 9A). The only anti-inflammatory cytokine that increased was IL-10 (Figure 9B). Several chemokines, including IP-10 (CXCL10), CCL2 (MCP-1), CCL3 (MIP-1α), CCL4 (MIP-1β), CCL5 (RANTES), and CCL7 (MCP-3) were also significantly increased upon infection (Figure 9C). Other pro-inflammatory and anti-inflammatory cytokines and chemokines that were measured and which did not significantly change in response to infection are shown in Supplementary Figure S4.

## Discussion

In this study we sought to characterize the mechanisms underpinning anemia in a rodent model of acute malaria, including whether optimal pro-inflammatory responses can be mounted to fight infection and whether this impacts on the erythrocyte compartment. Infection of Balb/c mice with *P. berghei* led to the development of anemia by 5 dpi, with both blood hemoglobin levels and hematocrit ∼60% that of uninfected mice, and declining even further to ∼50% by 8 dpi, underlining the substantial hemolysis of circulating RBC elicited by this pathogen (Evans et al., 2006; Lamikanra et al., 2007). The infection also led to a striking decrease in bone marrow cellularity (∼75% reduction), which was consistent with other rodent infection studies (Rencricca and Coleman, 1979; Maggio-Price et al., 1985; Asami et al., 1992). At 5 dpi, there was a significant decline in the proportion of BFU-E in the bone marrow, although the proportion of CFU-Es was still maintained, indicating that the BFU-Es were capable of differentiating into CFU-E at this time. By 8 dpi, however, the proportion of BFU-E in the bone marrow had increased, but the proportion of CFU-E had significantly declined and although the proportion of erythroblasts increased at this time as RBCs (and reticulocytes) entered the circulation, this was insufficient to compensate for the anemia as the hematocrit did not recover. The impact of *Plasmodium* infection may, therefore, be analogous to cases of chronic erythroid stress including hemolysis, where low numbers of CFU-E progenitors results in insufficient production of RBCs and consequential triggering of BFU-E production (Dolznig et al., 2006). In addition, there were clear perturbations to steady-state erythropoiesis in the bone marrow of infected mice, with the differentiation of the early erythroblasts into more mature erythroblasts impaired. The most profound defect was at the polychromatic and orthochromatic erythroblast stages, which are the stages when hemoglobin production peaks (Granick and Levere, 1964). A similar defect in erythroblast maturation was also observed in a *Plasmodium chabaudi* rodent infection study (Chang and Stevenson, 2004).

The reduction in bone marrow cells was not restricted to the erythroid lineage since the total number of cells within the megakaryocyte, monocyte and neutrophil lineages were also decreased upon infection. This was also reflected in decreased numbers of myeloid progenitor cells. The overall hematopoietic changes in the bone marrow may stem from several mechanisms. These include reduced proliferative capacity (for example, in response to inflammatory signals) (King and Goodell, 2011) and/or decreased survival of progenitors, both of which are known to be triggered by the production of hemozoin from hemoglobin by parasite infection (Lamikanra et al., 2009). However, mobilization of mature RBCs into the blood in response to the decline in hematocrit, monocytes, and neutrophils (and other innate immune cells) to relevant tissues in direct response to the infection, and progenitors to other sites, particularly the spleen, would also likely be significant factors (Hoggatt and Pelus, 2011).

Indeed, spleen cellularity increased as the infection progressed, consistent with the observed splenomegaly that is a hallmark of *Plasmodium* infection (CDC, 2019). Increased numbers of BFU-E, CFU-E and erythroblasts were observed at 5 dpi. The additional BFU-Es may have migrated from the bone marrow to the spleen in response to emergency erythropoiesis (Goris et al., 1990), but spleen-resident stress erythroid progenitor cells (called stress BFU-E) (Lenox et al., 2005) likely also account for the increase in CFUs and erythroblasts observed in the spleen. However, despite initial increases in BFU-E and CFU-E numbers in the spleen, by 8 dpi they were significantly decreased. This coincided with an increase in the proportions of erythroblast and reticulocyte proportions, indicating enhanced differentiation into more mature erythroid cells, presumably to compensate for declining RBC numbers in circulation. However, steady-state erythropoiesis was also perturbed in this organ in a similar manner to that observed in the bone marrow, with the differentiation of the early erythroblasts into more mature erythroblasts significantly affected at 8 dpi.

The numbers of splenic myeloid progenitor cells were also increased in *P. berghei*-infected mice. Myeloid restricted progenitor cells have been shown to migrate to the spleen in response to a *Plasmodium chaubaudi* infection, a response that was dependent on IFN-γ signaling (Sponaas et al., 2009; Belyaev et al., 2013). We also observed an increase in monocyte/macrophages and neutrophil numbers, which is in keeping with the important role played by macrophages in removing pRBC and uninfected RBC that circulate through the spleen (Evans et al., 2006). The significant decrease in the proportion of mature RBCs in the spleen is likely to be attributable to their mobilization into the blood.

Taken together, our findings are consistent with the findings summated from a diverse variety of human and rodent studies utilizing different *Plasmodium* species. For example, those that have shown reduced BFU-E and CFU-E numbers in the bone marrow of infected mice (Maggio-Price et al., 1985), suppressed erythropoiesis in the spleen and bone marrow during the early stages of infection (using microarray analysis) (Sexton et al., 2004), lower numbers of BFU-E in the bone marrow of *P. falciparum*-infected children from Gambia who exhibited moderate to severe malaria (Abdalla and Wickramasinghe, 1988) and suppressed *in vitro* development of CD34^+^/erythroid progenitor cells following exposure to *P. vivax* (Panichakul et al., 2012). Collectively this suggests similar underlying mechanisms, thereby validating the *P. berghei* mouse infection model.

Using a GFP-expressing parasite line, we demonstrated that no significant infection of erythroblasts occurred, ruling this possibility out as a major contributor to disrupted erythroblast maturation. Similarly, low levels of the erythropoiesis-stimulating cytokine EPO were not responsible; on the contrary, serum EPO levels actually increased during the infection, consistent with the higher EPO levels observed after infection of mice with *P. chabaudi* (Chang and Stevenson, 2004) and in several human studies examining patients with *P. falciparum* anemia (Burchard et al., 1995; Verhoef et al., 2002). We extended our studies to examine the EPO-responsiveness of erythroblasts by analyzing the activation of STAT5, which lies downstream of the EPO receptor (Damen et al., 1995), and show for the first time that this was not significantly affected. Whether downstream responses of pSTAT5 were impaired in erythroblasts cannot be ruled out. However, other studies have shown that expression of the essential erythroid-promoting transcription factor GATA1 was reduced in the bone marrow of *P. berghei*-infected C57/Bl6 mice (Sexton et al., 2004) and in human erythroid cells co-cultivated with hemozoin-laden human monocytes, with the erythroid cells also exhibiting reduced expression of the receptors for EPO and transferrin (Skorokhod et al., 2010). Indeed it has been suggested that defects in erythroblast maturation during infection may stem from transferrin receptor expression shifting from Ter119^+^ cells to non-erythroid cells (Chang and Stevenson, 2004), which would in turn affect the ability of erythroblasts to take up iron and thus synthesize the hemoglobin required for erythroblast maturation, however, this was not measured in our study. Iron availability for erythropoiesis is likely to be separately limited as hepcidin is known to be upregulated in malaria infection (Howard et al., 2007; Wang et al., 2011).

A common myeloid progenitor cell yields erythroid cells and megakaryocytes via a megakaryocyte-erythroid progenitor (MEP) as well as neutrophils and monocytes/macrophages via a granulocyte-macrophage progenitor (GMP). The observed perturbation to the erythroid lineage in the bone marrow of the *P. berghei*-infected mice most likely occurred at the level of the MEP since the proportion of both Ter119^+^ erythroid cells and CD61^+^ megakaryocyte cells declined, whilst that of the CD11b^+^Gr1^+^Ly6G^–^ monocytes and CD11b^+^Gr1^+^Ly6G^+^ neutrophils were significantly increased at 8 dpi. Cells of the myeloid and lymphoid lineage release inflammatory cytokines and chemokines that have been shown to contribute or associate with anemia (Kern et al., 1989; Lyke et al., 2004; Ong’echa et al., 2008). Infected mice showed an increase in TNF-α which has previously been shown to inhibit all stages of erythropoiesis (Dufour et al., 2003), and IFN-γ, which has been shown to upregulate the expression of multiple members of the TNF superfamily responsible for inhibiting erythroid growth and differentiation (Miller et al., 1989; Felli et al., 2005). In addition, IFN-γ activation of macrophages can also lead to increased hemophagocytic activity in the spleen, resulting in the loss of erythrocytes via endocytosis (Zoller et al., 2011). Increases were also observed in the levels of IL-6, IL-10, IL-18, IL-23, and RANTES. Interestingly, IL-6 induces hepcidin, which negatively affects erythropoiesis by inducing iron deficiency (Nemeth et al., 2004), whereas cytokines such as IL-18 and IL-23 (which are produced by monocytes and/or macrophages in addition to other cells) increase IFN-γ production (Nakamura et al., 1989). Although the IL-17 levels in the mice were too low to determine whether the increase upon infection was indeed significant, this cytokine, which is produced by Th17 cells in response to IL-23, induces chemokines and IL-6 and acts in concert with TNF-α to induce inflammation (McGeachy et al., 2019) and has been shown to be crucial for maintenance of splenic macrophages in *P. berghei*-infected mice (Ishida et al., 2013). Thus, taken together the pathophysiology of malaria-associated anemia overlaps to some degree with anemia of chronic disease, a cytokine-mediated anemia (Means, 1995; Weiss and Goodnough, 2005). Increases were also seen in the levels of the chemokines CCL2 (MCP-1), CCL3 (MIP-1α), CCL4 (MIP-1β), and CCL7 (MCP-3), which are all potent monocyte chemoattractants and activators, suggesting that monocyte/macrophages are central to these processes. Malaria is not the only infectious disease that leads to bone marrow disruption and depletion of progenitor cells; inflammatory mediators liberated in response to other pathogens can also have indirect effects on bone marrow progenitor cells, in addition to effects caused by direct infection of progenitor cells or recognition of the pathogen by these cells. Examples include chronic infections with human immunodeficiency virus and *Mycobacterium avium* and infections with the tick-borne pathogen *Ehrlichia muris* and the parasite *Toxoplasma gondii* (Glatman Zaretsky et al., 2014).

Notably, the cytokine and chemokine responses observed in our study are consistent with other studies. For example, injection of hemozoin into Balb/c mice led to the upregulation of transcripts encoding IL-6 and chemokines CCL2, CCL3, and CCL4 (Jaramillo et al., 2004). In addition, levels of IL-23 were higher in the peripheral blood of Kenyan children with malaria anemia (Ong’echa et al., 2008), whilst IL-18 promoter haplotypes that result in elevated IL-18 expression during acute infection have been associated with increased risk of SMA (Anyona et al., 2011). Gabonese children with severe malaria were found to have elevated levels of CCL3 and CCL4 in their circulation (Ochiel et al., 2005). Malian children with SMA showed elevated plasma IL-10 levels relative to healthy controls (Lyke et al., 2004). However, a low IL-10 to TNF-α ratio is associated with enhanced severity to malaria anemia (Othoro et al., 1999) and reduced serum IL-10 was observed in pediatric patients from Ghana with SMA (Kurtzhals et al., 1998). Other studies have also indicated a low concentration of serum RANTES is associated with anemia (Were et al., 2006, 2009). Finally, IL-12 and MIF have also been implicated in the pathophysiology of anemia (Martiney et al., 2000; Chaiyaroj et al., 2004) but we did not observe a significant increase in IL-12, nor did we measure levels of MIF.

In summary, the results from this study point to the acute anemia resulting from a *P. berghei* infection being due, at least in part, to direct hemolysis and RBC destruction but also the release of pro-inflammatory cytokines and chemokines that serve to inhibit erythroid development. It should be noted that the identity of the cells responsible for liberating the inflammatory cytokines and chemokines during infection were not analyzed as part of this study. Based on the changes to the myeloid numbers and increase in cytokines and chemokines that are known to be macrophage chemoattractants and activators, monocytes are likely to play a central role, serving as a defense mechanism to control the infection and giving rise to a pathophysiology with similarities to anemia of chronic disease. By defining the hematopoietic changes that occur during a *Plasmodium* infection in our validated model, this will complement human studies and help to gain a deeper understanding on the pathophysiology of anemia. For example, the five single nucleotide polymorphisms in the human Cytokine-inducible Src homology 2 domain-containing protein (CISH) gene (three of which lie in the promoter region) that have been associated with increased risk to malaria (Khor et al., 2010) can now be experimentally validated for the contribution to malaria anemia using *Cish* knockout mice infected with *P. berghei*.

## Data Availability Statement

All datasets generated for this study are included in the article/Supplementary Material.

## Ethics Statement

The animal study was reviewed and approved by the Deakin University Animal Welfare Committee.

## Author Contributions

AL, RL, and HD conducted the experiments. AL, AW, and TK-W designed the experiments. AL, AW, and TK-W wrote the manuscript. All authors read the manuscript and approved the final version.

## Conflict of Interest

The authors declare that the research was conducted in the absence of any commercial or financial relationships that could be construed as a potential conflict of interest.

## Footnotes

1 http://rsbweb.nih.gov/ij

## Supplementary Material

FIGURE S1 Representative FACS plots of erythroblasts isolated from the bone marrow and spleen of mice at different stages of infection. Ter119^+^ cells in the bone marrow **(A)** and spleen **(B)** at distinct stages of erythroid differentiation were identified and quantified based on the gating strategy outlined in Figure 1. Representative FACS blots of cells harvested from mice at 0, 5, and 8 dpi are shown. Pro, proerythroblasts; Baso, basophilic erythroblasts; Poly, polychromatic erythroblasts; Ortho, orthochromatic erythroblasts; Retics, reticulocytes; RBC, red blood cells.

FIGURE S2 Parasitism of erythroblasts by *P. berghei*. Erythroblasts (CD44^+^Ter119^+^) from the spleen of mice infected with *P. berghei* EXP2-FRT-HA parasites expressing a GFP reporter were analyzed for GFP expression by FACS **(A)**. Erythroblasts (CD44^+^Ter119^+^) were also sorted by FACS, stained with DAPI (to stain the nucleus) and analyzed for GFP expression by fluorescence microscopy **(B)**. *P. berghei*-infected (Pb) RBC and uninfected erythroblasts served as negative controls, whilst *P. berghei*-GFP infected reticulocytes served as positive controls for GFP expression. Scale bar 5 μm.

FIGURE S3 Representative FACS plots of CD61^+^ megakaryocytes, neutrophils, and monocytes isolated from the bone marrow and spleen of mice at different stages of infection. The numbers of megakaryocytes (CD61^+^, Gr1^–^), neutrophils (Gr-1^+^, Ly6G^+^), and monocytes (Gr-1^+^, Ly6G^–^) in the bone marrow **(A)** and spleen **(B)** were identified and quantified based on the gating strategies outlined in Figure 6. Representative FACS blots of cells harvested from mice at 0, 5, and 8 dpi are shown.

FIGURE S4 Pro-inflammatory and anti-inflammatory cytokines and chemokines that were not significantly altered in the serum during *P. berghei* infection. Levels of the indicated proinflammatory cytokines **(A)**, anti-inflammatory cytokines **(B)**, and chemokines **(C)** that did not significantly change in the serum of infected mice at 7 dpi. The lower limits of detection are indicated by a dotted line. Data represents results from individual mice as well as the mean ± SD (*n* = 6 mice). An unpaired *t*-test was used to calculate statistical significance.
