# Supplementary figures and images for: Acute Plasmodium berghei Mouse Infection Elicits Perturbed Erythropoiesis With Features That Overlap With Anemia of Chronic Disease
Source: Front Microbiol. 2020 Apr 16;11:702. doi: 10.3389/fmicb.2020.00702 (PMC7176981; doi:10.3389/fmicb.2020.00702)

FIGURE S1

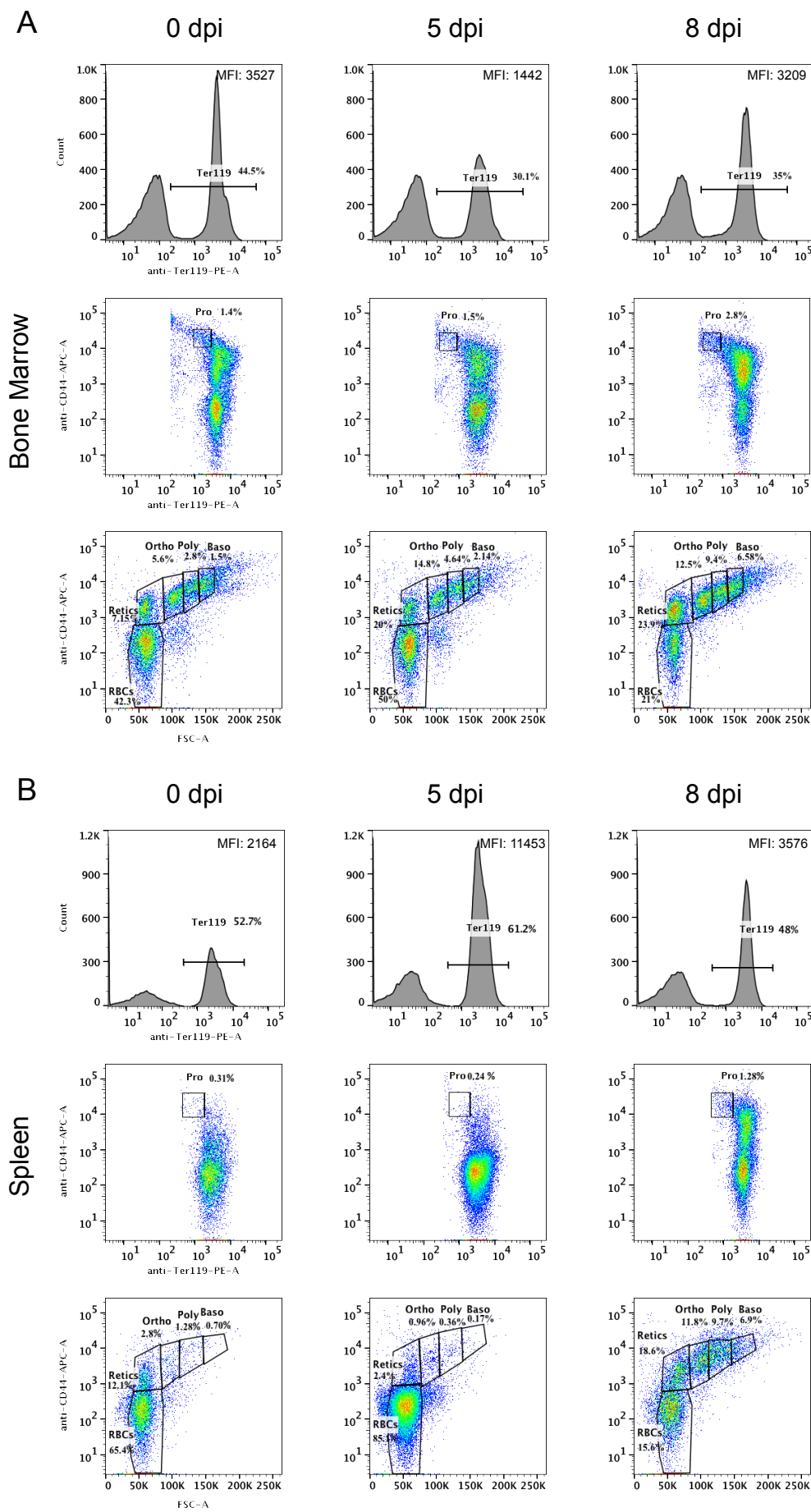

FIGURE S2

A

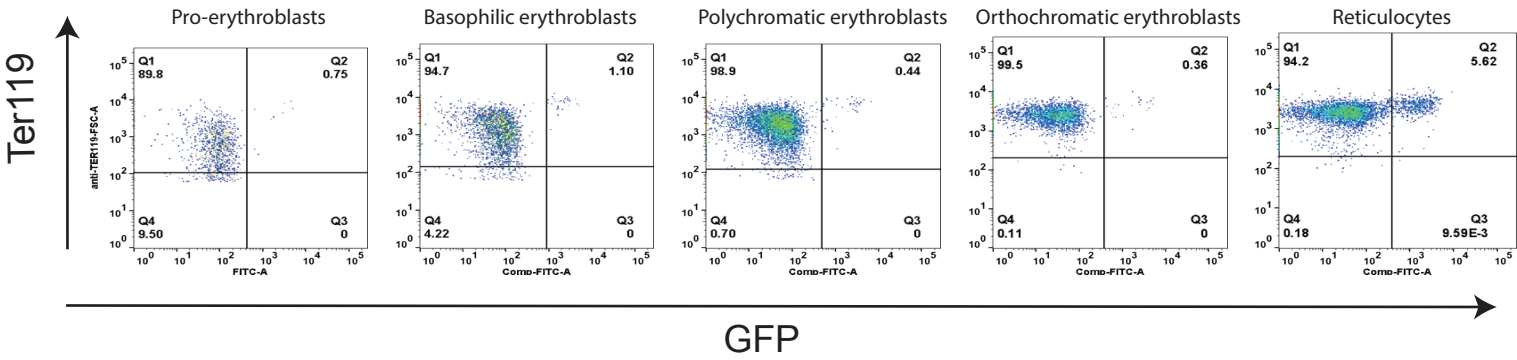

B

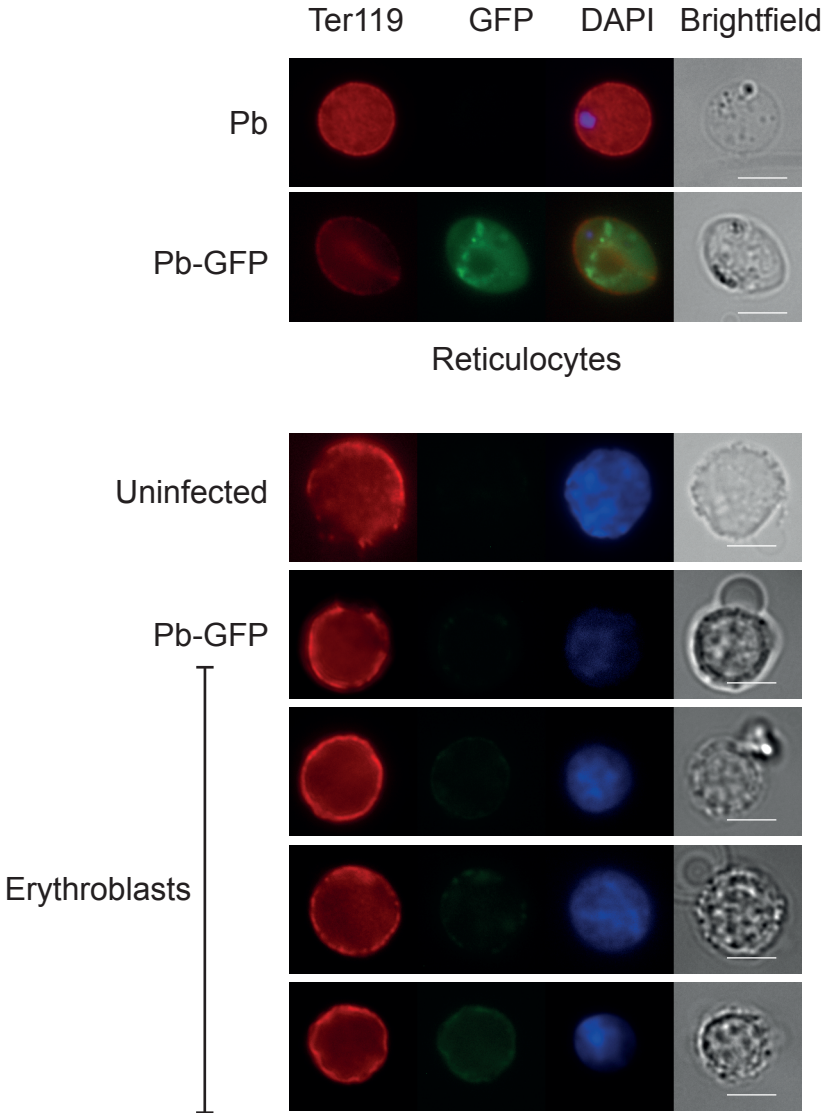

FIGURE S3

A

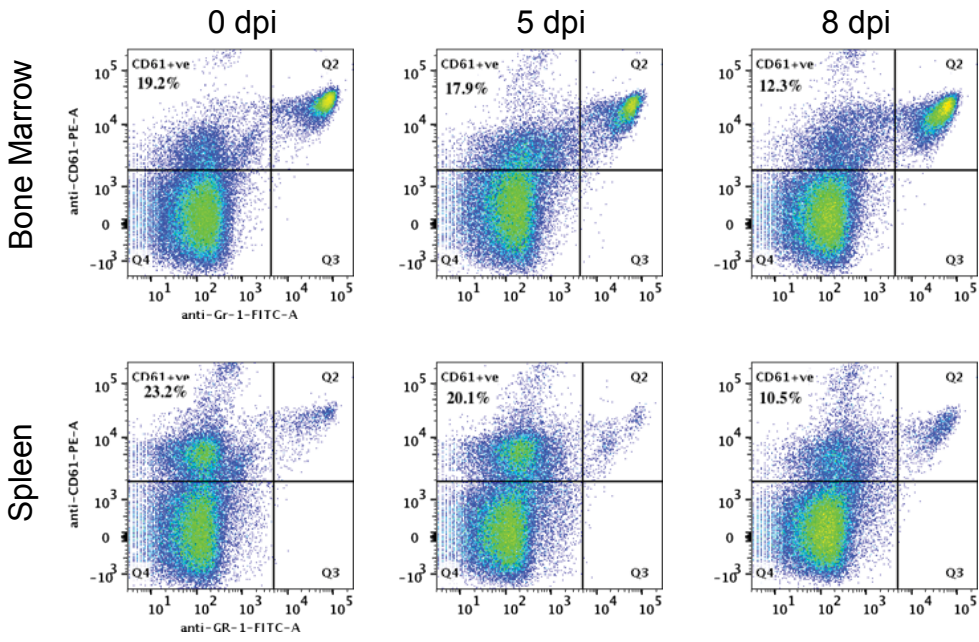

B

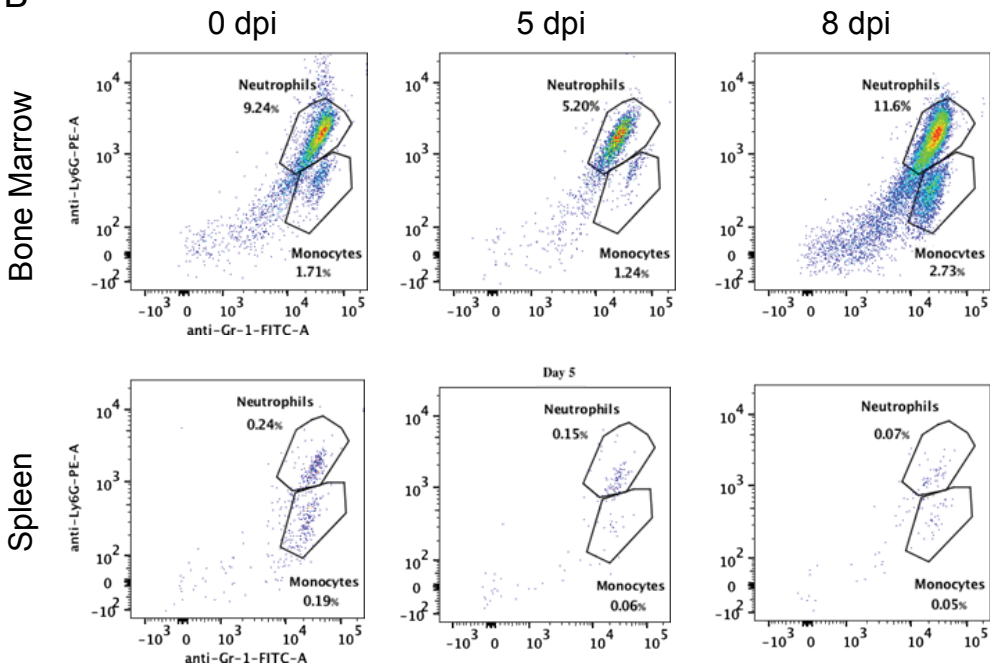

FIGURE S4

A

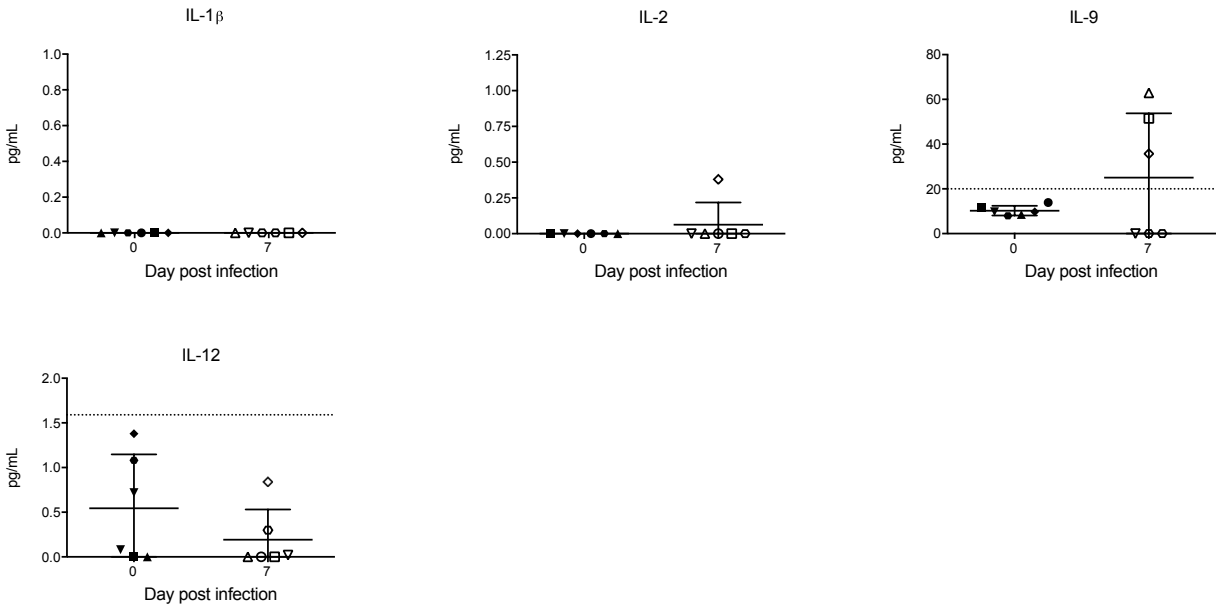

B

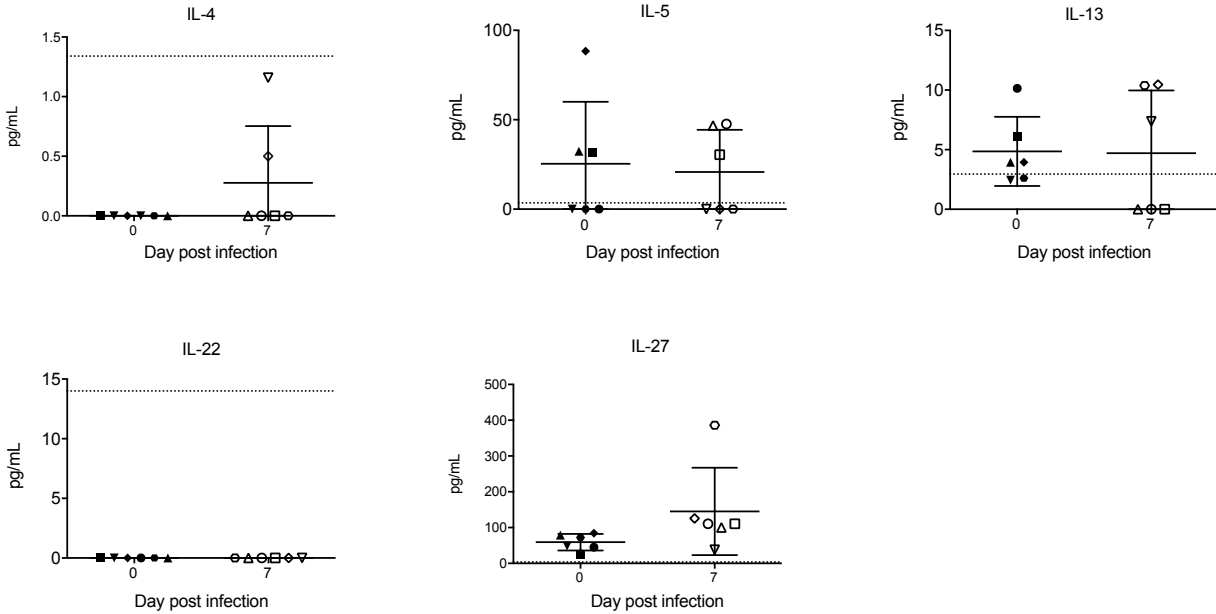

C

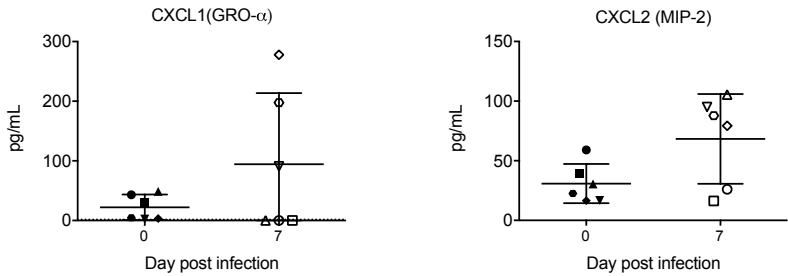

Supplement: FIGURE S1 — Representative FACS plots of erythroblasts isolated from the bone marrow and spleen of mice at different stages of infection. Ter119+ cells in the bone marrow (A) and spleen (B) at distinct stages of erythroid differentiation were identified and quantified based on the gating strategy outlined in Figure 1. Representative FACS blots of cells harvested from mice at 0, 5, and 8 dpi are shown. Pro, proerythroblasts; Baso, basophilic erythroblasts; Poly, polychromatic erythroblasts; Ortho, orthochromatic erythroblasts; Retics, reticulocytes; RBC, red blood cells. [file Data_Sheet_1.pdf]
